# Giant Retroperitoneal Mass During Pregnancy With an Unexpected Diagnosis: Ovarian Cyst or Something Else?

**DOI:** 10.7759/cureus.92057

**Published:** 2025-09-11

**Authors:** Maria J Guaman, Alejandra Vargas, Isis X Muñoz, Andrea G Ramirez, Melanie Caballero Garcia, Rommel F Portilla, Jose L Guaman

**Affiliations:** 1 Faculty of Medicine, Universidad Católica Boliviana "San Pablo", Santa Cruz de la Sierra, BOL; 2 Department of General Medicine, Universidad Privada San Juan Bautista, Lima, PER; 3 Faculty of Medicine, Universidad Nacional Autónoma de México, Mexico City, MEX; 4 Faculty of Medicine "Alberto Hurtado", Universidad Peruana Cayetano Heredia, Lima, PER; 5 Department of General Medicine, Universidad de San Martín de Porres, Lima, PER; 6 School of Medicine, Universidad de las Américas, Quito, ECU; 7 Department of Obstetrics and Gynecology, Hospital de la Mujer Dr. Percy Boland Rodriguez, Santa Cruz, BOL

**Keywords:** adrenal hydatid cyst, echinococcus granulosus (e. granulosus), giant abdominal mass, hydatid disease in pregnancy, misdiagnosed ovarian cyst, multidisciplinary surgical approach, retroperitoneal cyst during pregnancy

## Abstract

Abdominal masses during pregnancy are uncommon and challenging to diagnose. Anatomical changes and imaging limitations often complicate evaluation. We present the case of a 30-year-old woman at 30 weeks of gestation who was admitted with progressive abdominal distension and discomfort. Initial ultrasound suggested a large ovarian cyst, but further imaging revealed a giant retroperitoneal mass. An exploratory laparotomy identified a cystic lesion over 25 cm in size containing approximately nine liters of citrine fluid. Partial resection was performed with preservation of maternal stability, and histopathology confirmed a benign suprarenal hydatid cyst. Postoperative recovery was favorable, and the pregnancy progressed without complications. At 39 weeks of gestation, the patient underwent cesarean section and delivered a healthy neonate. Both mother and child remained stable and were discharged in good condition. This case illustrates the importance of considering retroperitoneal pathology in the differential diagnosis of abdominal masses during pregnancy. Hydatid disease, although rare, should be considered in patients from endemic regions, particularly when imaging findings are atypical. Surgical management during pregnancy, while complex, can be safe and effective when conducted in an experienced, multidisciplinary setting.

## Introduction

Abdominal masses during pregnancy are uncommon findings. Among them, adnexal masses have an estimated incidence of approximately 1% of pregnancies, and most resolve spontaneously [1]. In contrast, retroperitoneal masses are even rarer in the general population and are usually detected incidentally due to their silent growth and nonspecific symptoms. During pregnancy, their diagnosis is further complicated by anatomical distortion and the potential for misidentification as adnexal lesions [2].

Among the various etiologies of retroperitoneal masses, retroperitoneal cysts constitute a heterogeneous group of lesions, including lymphangiomas, pseudocysts, and parasitic infections such as hydatid disease. These cysts may grow substantially before becoming clinically evident and, during pregnancy, can cause uterine displacement and compression of adjacent organs, potentially resulting in maternal and fetal complications [3,4].

This report presents the case of a 30-year-old pregnant woman at 30 weeks of gestation, in whom an abdominal mass initially interpreted as a giant ovarian cyst was intraoperatively found to be a large retroperitoneal cyst arising from the adrenal gland. Histopathological analysis confirmed its benign nature, consistent with a hydatid cyst. The aim of this study is to describe the diagnostic and therapeutic approach to this rare entity during pregnancy, as well as to highlight the importance of comprehensive evaluation and coordinated management in complex clinical settings involving both maternal and fetal well-being.

## Case presentation

A 30-year-old pregnant woman at 30 weeks of gestation was referred to the emergency department with progressive abdominal distension associated with a sensation of heaviness, early satiety, and diffuse abdominal discomfort. The patient also reported increasing difficulty with ambulation, mild dyspnea on exertion, and intermittent lumbar pain over the preceding weeks. There was no history of fever, gastrointestinal or urinary symptoms, vaginal bleeding, prior surgical procedures, or known gynecologic conditions.

Initial obstetric ultrasound confirmed a viable singleton intrauterine pregnancy. Additionally, a large cystic mass was identified in the right adnexal region, initially suggestive of a giant ovarian cyst. Given the limitations of ultrasound in clearly defining the lesion's origin, an abdominopelvic computed tomography (CT) scan was performed. While magnetic resonance imaging (MRI) is generally the modality of choice during pregnancy because it avoids ionizing radiation, MRI was not feasible in this case. The decision was influenced by two key factors: the limited availability of MRI within the public healthcare system and the patient's socioeconomic constraints, since MRI carries a significantly higher cost not covered by the National Universal Health Insurance program.

Following comprehensive counseling regarding diagnostic alternatives, the patient received detailed information about the potential fetal radiation risks associated with CT. Particular emphasis was placed on the fact that, although CT involves ionizing radiation, the radiation dose from a single abdominopelvic study is considered low and unlikely to produce teratogenic effects. Nonetheless, the small theoretical risk of long-term consequences, such as an increased risk of childhood malignancy, was acknowledged and discussed. After weighing these risks against the clinical need for an accurate and timely diagnosis, the patient provided informed consent and opted for the examination.

**Figure 1:**
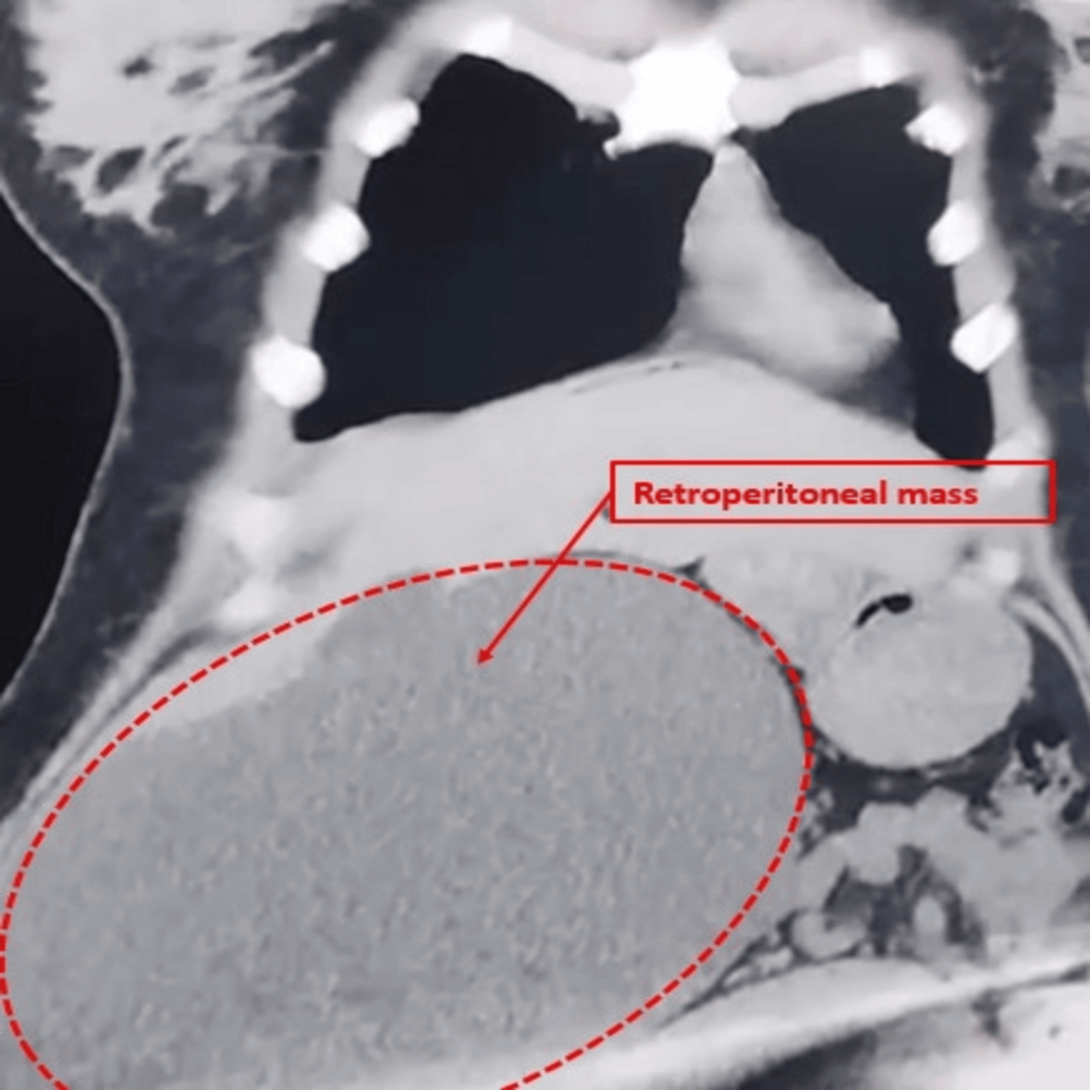
Coronal CT scan showing a giant retroperitoneal cystic mass Coronal non-contrast CT scan of the abdomen revealing a large, well-circumscribed hypodense retroperitoneal cystic mass (outlined with a red dashed line). The lesion occupies the right flank, displacing the right kidney medially and superiorly. No internal septations, solid components, or calcifications are visible. These findings are consistent with a retroperitoneal cystic lesion; differential diagnoses include hydatid cyst, lymphangioma, or adrenal pseudocyst. CT: computed tomography

The CT scan demonstrated a large cystic mass in the lower right quadrant, without evidence of torsion, rupture, or malignant features. As shown in Figure 1, the lesion appeared as a well-circumscribed, hypodense retroperitoneal cyst, displacing the right kidney medially and superiorly.

**Figure 2:**
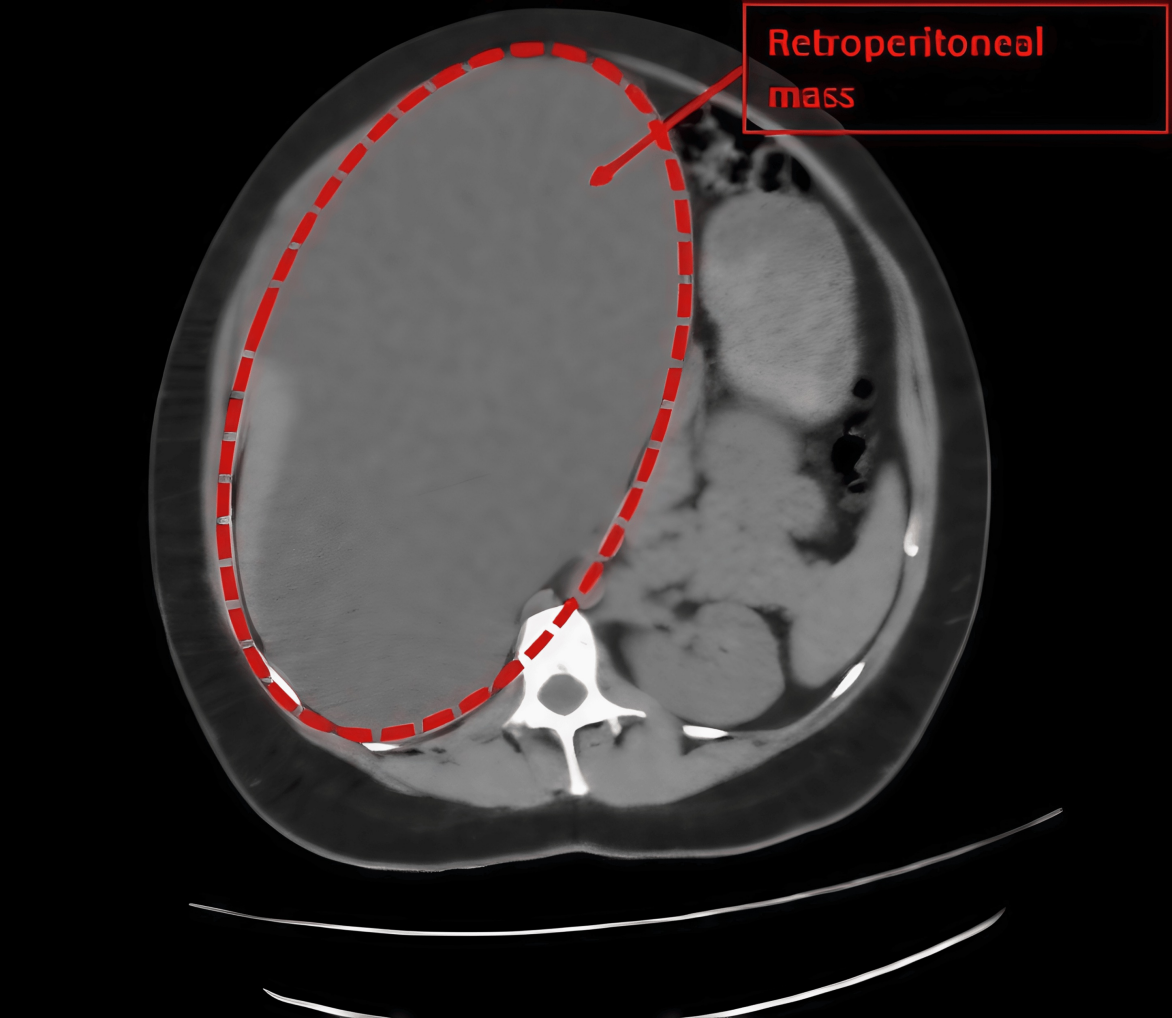
Axial CT scan demonstrating a large retroperitoneal cystic lesion Axial non-contrast abdominal CT image showing a large, well-defined hypodense cystic mass located in the right retroperitoneal space (outlined with a red dashed line). The lesion causes medial displacement of the right kidney and exerts mass effect on adjacent abdominal structures. No internal septations, mural nodules, or calcifications are observed. These imaging characteristics are suggestive of a retroperitoneal cystic lesion. CT: computed tomography

Figure 2 provides an axial view that further demonstrates the lesion's size and compressive effect on adjacent abdominal organs.

Due to the risk of compression of intra-abdominal structures, including the gravid uterus, with potential maternal and fetal compromise, exploratory laparotomy was indicated. The decision was made following evaluation and consent from a multidisciplinary team composed of gynecology, general surgery, and anesthesiology.

Perioperative antibiotic prophylaxis with ceftriaxone and metronidazole was administered to minimize the risk of intra-abdominal infection and chorioamnionitis. Particular care was taken to mitigate fetal risks: maternal hemodynamics were closely monitored to avoid hypotension, anesthesia was tailored to pregnancy safety standards, continuous intraoperative fetal heart rate monitoring was maintained by the obstetric team, and tocolytic therapy with nifedipine was administered to reduce the risk of uterine contractions and preterm labor.

Intraoperatively, a giant retroperitoneal mass was identified adjacent to the right adnexa, markedly displacing the uterus and neighboring abdominal organs, with no continuity to the ovary or adnexal structures. The lesion measured approximately 25.3×25.1×28 cm, with an estimated volume of 8,295 cm³. A controlled incision of the cyst capsule was performed, draining nearly nine liters of clear, citrine fluid. Subsequent dissection enabled partial mobilization and resection of approximately 95% of the cyst. Complete excision was not feasible due to intimate adherence to the duodenum and major vessels; therefore, the residual wall in this region was ligated to reduce operative risk. Although intraoperative frozen section was considered, it was not pursued due to institutional economic limitations. A Hemovac drain was placed, uterine integrity was preserved, and the procedure was completed uneventfully with no evidence of bleeding or fetal compromise. The essential intraoperative details, including cyst characteristics and maternal-fetal outcomes, are outlined in Table 1.

**Table 1:** Intraoperative findings and surgical management

Parameter	Description
Location	Retroperitoneal, adjacent to the right adnexa; uterus and abdominal organs displaced; no continuity with the ovary/adnexa
Size	25.3×25.1×28 cm (estimated volume: 8,295 cm³)
Capsule management	Controlled incision with drainage of ~9 liters of clear, citrine fluid
Extent of resection	Partial resection (~95%); complete excision not feasible due to adherence to the duodenum and major vessels
Residual management	Residual wall ligated at the duodenal region to minimize operative risk
Frozen section	Considered but not performed due to institutional economic limitations
Drain placement	Hemovac drain inserted into the surgical cavity
Maternal-fetal outcome	Uterine integrity preserved; no bleeding or fetal compromise; procedure completed uneventfully

Histopathological analysis revealed a cyst wall with an acellular laminated layer consistent with a suprarenal origin, associated with congestive adipose tissue and no evidence of atypia or malignancy. However, a gross specimen photograph was not obtained, and histopathological photomicrographs were unavailable because the specimen was processed in a private laboratory, which only provided the written report. Serological testing for *Echinococcus granulosus*, performed postoperatively, was initially negative. Given the unusual findings, the test was repeated to rule out a false-negative result and subsequently returned positive. In correlation with the imaging features, the case was ultimately confirmed as a retroperitoneal hydatid cyst of adrenal origin.

Postoperative recovery was uneventful, with satisfactory maternal progress. At short-term follow-up, no complications were noted, and fetal growth remained appropriate. Given the history of major abdominal surgery (exploratory laparotomy at 30 weeks of gestation), cesarean delivery was performed at 39 weeks, as determined by the last menstrual period, to optimize maternal-fetal safety and reduce potential risks associated with labor. A live neonate was delivered with Apgar scores of 7 and 8 at one and five minutes, respectively. Following delivery, antiparasitic therapy with albendazole was initiated to reduce the risk of recurrence. The patient has been referred for long-term follow-up, including periodic imaging and repeat serological testing, which remain ongoing at the time of this report.

## Discussion

The management of retroperitoneal masses during pregnancy is exceptionally challenging due to their rarity and silent growth and the anatomical distortion caused by the gravid uterus, which limits precise imaging characterization. While hydatid cysts in atypical locations have been reported, diagnosis is often delayed until compressive symptoms arise or incidental findings appear on obstetric ultrasound. This underscores the importance of comprehensive evaluation, prioritizing maternal and fetal health, and also multidisciplinary coordination involving gynecology, general surgery, radiology, and anesthesiology to determine optimal timing and treatment.

Recent reports illustrate the heterogeneity of presentation and outcomes. Kiemtoré et al. [5] described a 42 cm serous cystadenoma diagnosed postpartum and managed with laparotomy, with the patient delivering vaginally without complications. Sinha et al. [6] reported a massive mucinous cystadenoma complicating a term pregnancy, safely treated after delivery, emphasizing the role of prenatal surveillance. By contrast, Brezeanu et al. [7] presented a first-trimester adnexal hydatid cyst requiring surgical removal and cesarean delivery due to fetal distress. Wen et al. [3] documented a retroperitoneal mucinous cyst in a term pregnancy, while Clegg et al. [8] described a mature retroperitoneal teratoma during gestation, both highlighting the diagnostic challenge of distinguishing adnexal from retroperitoneal lesions.

Hydatid disease during pregnancy remains extremely rare, with most cases involving the liver or lungs and retroperitoneal or adrenal involvement being exceptional. Tajmalzai and Aien [9] recently reviewed adrenal hydatid cysts, reinforcing their rarity and diagnostic pitfalls. Importantly, a 2023 review by Al Jumaah et al. identified fewer than 57 adrenal cases in the global literature, none during pregnancy [10]. To our knowledge, our report is among the very few describing an adrenal-origin retroperitoneal hydatid cyst in a pregnant woman, underscoring its novelty. The absence of the typical "double-wall sign", combined with an initially negative postoperative serology that later turned positive on repeat testing, further complicated the diagnosis and highlights the importance of correlating imaging, intraoperative findings, and epidemiological context.

MRI, although not available in this case due to resource limitations, would have been preferable for lesion characterization in pregnancy. It offers superior soft-tissue resolution without ionizing radiation and is supported by recent guidelines as safe for pregnant patients [11]. In selected scenarios, MRI may enable the earlier recognition of atypical features and facilitate surgical planning.

In our case, surgery was indicated at 30 weeks of gestation because of the high risk of uterine and visceral compression with potential maternal-fetal compromise, including respiratory distress, vascular compression, and preterm labor. A multidisciplinary team coordinated the intervention, and partial resection (~95%) was achieved with preservation of uterine integrity and favorable maternal-fetal outcomes. This makes the present case unique in the literature, both for its adrenal origin and for successful surgical management during an ongoing pregnancy.

## Conclusions

This case underscores the diagnostic complexity of abdominal masses during pregnancy, where physiological changes and imaging limitations can obscure the anatomical origin of lesions. Retroperitoneal hydatid disease of suprarenal origin represents an exceptionally rare entity, but it should remain within the differential diagnosis in patients from endemic regions when imaging findings are inconclusive. Awareness of this possibility is essential to avoid misinterpretation as adnexal or neoplastic pathology. Surgical management during pregnancy, although technically challenging, can achieve favorable outcomes when guided by multidisciplinary coordination and vigilant obstetric care. More broadly, this case highlights the importance of integrating epidemiological context into diagnostic reasoning and reinforces the need for timely recognition and intervention to optimize both maternal and fetal prognosis. Strengthening prenatal imaging protocols and clinical awareness in endemic regions may further improve early detection and facilitate appropriate surgical planning.
